# Fit for the Eye: Aptamers in Ocular Disorders

**DOI:** 10.1089/nat.2015.0573

**Published:** 2016-06-01

**Authors:** Daniel W. Drolet, Louis S. Green, Larry Gold, Nebojsa Janjic

**Affiliations:** SomaLogic, Inc., Boulder, Colorado.

## Abstract

For any new class of therapeutics, there are certain types of indications that represent a natural fit. For nucleic acid ligands in general, and aptamers in particular, the eye has historically been an attractive site for therapeutic intervention. In this review, we recount the discovery and early development of three aptamers designated for use in ophthalmology, one approved (Macugen), and two in late-stage development (Fovista and Zimura). Every one of these molecules was originally intended for other indications. Key improvements in technology, specifically with regard to libraries used for *in vitro* selection and subsequent chemical optimization of aptamers, have played an important role in allowing the identification of development candidates with suitable properties. The lessons learned from the selection of these molecules are valuable for informing us about the many remaining opportunities for aptamer-based therapeutics in ophthalmology as well as for identifying additional indications for which aptamers as a class of therapeutics have distinct advantages.

## Introduction

The promise of aptamers as a new class of therapeutics emerged concurrently with the discovery of systematic evolution of ligands by exponential enrichment (SELEX) [1,2], the method of identifying nucleic acid-based ligands from large, randomized nucleic acid libraries. In the early 1990s, this leap of faith was understandable since monoclonal antibodies were becoming broadly accepted as an important class of biologics and aptamers exhibited high affinity and specificity similar to antibodies. Some obvious advantages of aptamers, such as chemical synthesis, cost of goods, and *in vitro* evolution without the need for animals, provided added appeal for this notion. All of the challenges appeared readily solvable, especially since the parallel interest in antisense therapeutics meant that there would be sufficient critical mass of pharmaceutical scientists jointly motivated to solve common manufacturing and clinical development problems.

Twenty-five years on, there is one approved drug (Macugen/pegaptanib), one in phase 3 (Fovista/pegpleranib), and one in phase 2 development (Zimura/ARC1905), all intended for ophthalmic use. In this perspective, we will review the events that led to the discovery and early development of these three drug candidates, as well as the decisions to focus on diseases of the eye. We will also address the future of aptamer-based therapeutics in the context of steadily improving chemical versatility available for use in aptamers (as well as other nucleic acid therapeutics) and suggest the types of indications and opportunities for which aptamers may be the most competitive class of therapeutics in the eye.

## Libraries for SELEX

When considering development of aptamers as therapeutics, one of the most obvious challenges is nuclease resistance. With RNA, this was an enormous challenge, illustrated by the fact that scientists working with RNA needed to keep their benches in immaculate condition. The key breakthrough came from the laboratory of Fritz Eckstein, who showed that substitution of 2′-hydroxyl (2′-OH) groups with 2′-amino (2′-NH_2_) or 2′-fluoro (2′-F) groups at pyrimidines imparted a substantial degree of nuclease resistance, despite the fact that all of the purines remained unmodified [3]. The reason for this effect is due to the fact that the major ribonuclease (RNAse) in serum, RNAse A, has substrate specificity narrowly confined to pyrimidines.

Importantly, these modifications were compatible with all of the enzymatic steps of SELEX: both 2′-aminopyrimidine and 2′-fluoropyrimidine triphosphates were accepted as substrates by T7 RNA polymerase, and the corresponding modified transcripts could be converted into DNA by reverse transcriptases (amplification by polymerase chain reaction could then be done according to standard protocols) [4–7]. Because of their duplex stabilities and ease of synthesis, the use of such modified libraries, especially the 2′-fluoropyrimidine libraries, is still the preferred starting point of many SELEX experiments today.

## Vascular Endothelial Growth Factor as a Therapeutic Target

The potential of the technology was immense and we spent much of the early period selecting appropriate therapeutic targets. We decided to focus initially on extracellular targets since nucleic acids, being large and charged molecules, are not transported efficiently across the lipid-rich cell membranes (we could always revisit intracellular targets once the challenging problem of intracellular delivery was solved). This was a broad and impactful strategic decision that affected all of our therapeutic programs. Within this broad category, we became intrigued with the notion promoted by Judah Folkman and many others that inhibition of new blood vessel growth (angiogenesis) may be a powerful and novel method of treating many pathological conditions, including cancer, psoriasis, rheumatoid arthritis, and ocular disorders [8].

Many of the angiogenic growth factors such as basic fibroblast growth factor and vascular endothelial growth factor (VEGF) exert their biological effects by binding to specific receptors on cell surfaces—these receptors then transmit the signal into the cell interior and trigger a complex sequence of events that result in endothelial cell growth. Many also bind to heparin, a polyanion like nucleic acids, suggesting a natural fit as SELEX targets. Thus, aptamers could inhibit growth factor signaling by interfering with the first step in this cascade, receptor binding, without needing to enter into the cells—also a good match for our initial strategy [9,10].

The protein that would later become known as VEGF was discovered in the laboratory of Harold Dvorak at Beth Israel Hospital in Boston. Dr. Dvorak and his associates noticed that tissue samples from many tumors contained fibrin filaments outside of the blood vessels. Since fibrin is generally absent in normal tissues, Dr. Dvorak's team suspected that tumor cells were secreting a factor that was making blood vessels permeable to proteins normally confined within blood vessels. They soon succeeded in isolating such a protein and called it vascular permeability factor (VPF) [11,12].

Almost a decade later, several groups independently isolated a protein (named VEGF) that was capable of selectively inducing the growth of endothelial cells. It soon became clear that VPF and VEGF were one and the same protein [13–18]. The name VPF/VEGF was used in many publications initially, but the scientific community appears to have grown tired of using such an unwieldy acronym and VEGF is the name that stuck. Nevertheless, VEGF is the only angiogenic growth factor capable of inducing both vascular leakage and endothelial cell growth. The two activities are functionally related since leakage of proteins from blood vessels is believed to provide a provisional scaffold on which growing endothelial cells migrate to form nascent blood vessels [19].

By the time we began to consider suitable targets for SELEX, the suspicion that VEGF was a key inducer of angiogenesis was growing. In 1992, Eli Keshet at the Hebrew University demonstrated that hypoxia stimulated VEGF production by tumor cells [20]. Thus, tumor cells could respond to inadequate blood supply by producing an inducer of new blood vessel growth. In 1993, an article from Napoleone Ferrara's group at Genentech demonstrated that an inhibitory monoclonal antibody to VEGF reduced the rate of tumor growth in mice [21].

These exciting findings established VEGF as a key angiogenic growth factor in tumors. Later that year and reflecting the sentiment shared by many scientists in the field, Michael Klagsbrun and Shay Soker published an article entitled “VEGF/VPF: the angiogenesis factor found?” [22]. The mouse antibody described in the Genentech article was humanized and eventually became Avastin (bevacizumab). Avastin was initially approved by the Food and Drug Administration (FDA) in 2004 for the treatment of colon cancer in combination with 5-fluorouracil. As we will see later, Avastin is now also an important off-label treatment option for ocular disorders.

## VEGF Isoforms and Age-Related Macular Degeneration

**FIG. 1.**
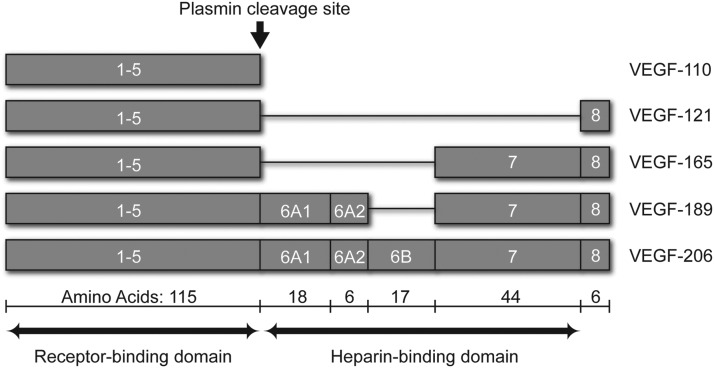
Exon structure of major vascular endothelial growth factor-A (VEGF-A) isoforms (adapted from Eming and Krieg [189]).

VEGF is a secreted disulfide-linked homodimer that selectively stimulates endothelial cells to proliferate, migrate, and produce matrix-degrading enzymes [13,14,17,23,24], all of which are required for the formation of new vessels. The biological effects of VEGF are mediated through two tyrosine kinase receptors, Flt-1 and Flk-1/KDR [also known as VEGF receptor 1 (VEGFR1) and VEGFR2], which are predominantly expressed not only on cells of endothelial origin [25–27] but also on other cell types [28]. VEGF occurs in several isoforms as a result of alternative splicing of the VEGF gene that consists of eight exons (Fig. 1) [28–30].

The most prevalent isoforms are VEGF-121, VEGF-165, VEGF-189, and VEGF-206 [28]. Exons 6 and 7 encode basic heparin binding domains and their alternative use determines the affinity of the isoform for heparin and therefore influences diffusion within tissues. With both basic exons, VEGF-189 and VEGF-206 bind to heparin with high affinity and remain largely cell associated. With only exon 7-encoded domain, VEGF-165 has intermediate affinity for heparin and is partially diffusible [29,31]. Without either of the exons, VEGF-121 is the only alternatively spliced isoform that does not bind to heparin and is readily diffusible; however, it appears to have a lower affinity for the VEGFRs [32] as well as reduced mitogenic potency [33].

Proteolytic processing of VEGF can generate additional isoforms. VEGF-165 can be cleaved by plasmin between Arg-110 and Ala-111 to generate VEGF-110, which is functionally equivalent to VEGF-121 [33]. VEGF-189 can be cleaved by urokinase within the exon 6 domain, and then further by plasmin, to generate VEGF-110 [34]. In addition, a subset of matrix metalloproteases (MMPs), especially MMP-3, −7, −9, and −19, is capable of cleaving VEGF-165 and VEGF-189 in sequential steps to generate VEGF-113, which is functionally equivalent to VEGF-110 [35]. Therefore, the relative abundance of matrix-bound and diffusible forms of VEGF in a given tissue is determined by the combination of alternative splicing and proteolytic processing [36]. Taken together, VEGF-165 is clearly both the most prevalent and the most potent isoform of VEGF. Thus, inhibition of VEGF-165 alone was plausibly sufficient to attenuate most pathological angiogenesis.

In addition to these observations, there was a justifiable concern at that time (late 1990s) that complete inhibition of all VEGF isoforms could result in undesirable adverse effects on the vascular system. The essentiality of VEGF and VEGFRs during embryonic development was established in gene deletion studies. Homozygous deletion of the VEGFR1 gene in mice results in embryonic lethal phenotype, with differentiated endothelial cells that organize into abnormal dilated vessels [37]. VEGFR1 appears to serve as a negative regulator of VEGF signaling in embryonic development since mice expressing only a truncated form of the receptor that lacks the intracellular kinase domain survive with the only apparent abnormality being impaired macrophage migration in response to VEGF stimulation [38].

The notion that partial attenuation of VEGF signaling is essential for proper development of the vasculature is further supported by the observation that overexpression of VEGF results in a phenotype similar to that of VEGFR1 knockout mice [38]. Alternatively spliced secreted form of VEGFR1 is a potent naturally occurring antagonist of VEGF [39]. VEGFR2 knockout mice also die *in utero*, with a more extensive disruption of the vasculature in which endothelial cells and hematopoietic cells (both derived from the hemangioblast) fail to develop [40,41].

In contrast to VEGFR1 and VEGFR2 heterozygous knockout mice, which are viable, deletion of even one allele of the VEGF gene is sufficient for embryonic lethality [42,43]. Mice that express only VEGF-120 are also not viable [31]. These observations illustrate the essential need for very tight regulation of VEGF signaling during development, with severe consequences associated with departure in either direction from the normal range.

In the nervous system, VEGF exerts a protective effect for several types of neurons [44]. VEGF is expressed by neurons and glial cells in the spinal cord, whereas VEGFR2 and neuropilin-1 (the VEGF-165 receptor) are expressed by motor neurons [45]. In contrast to heterozygous VEGF knockout mice that express about 50% of wild-type VEGF levels and die *in utero*, mice that express about 75% of wild-type levels due to targeted deletion of the hypoxia response element in the promoter region of the VEGF gene are viable. However, these so-called VEGF^δ/δ^ mice develop adult-onset motor neuronal defects resembling amyotrophic lateral sclerosis (ALS) [45]. Consistent with this observation are results from a genetic association study, which showed that Europeans with a similar haplotype in the promoter and leader sequences of the VEGF gene have reduced plasma levels of VEGF and are at a higher risk of developing ALS in adulthood [46]. Other than its effects on motor neurons, VEGF increases the survival of hippocampal, cortical, cerebellar granule, dopaminergic, autonomic, and sensory neurons and stimulates the growth and migration of astrocytes and glial cells [44].

In the eye, VEGF is a survival factor for retinal neurons [47] and plays an essential role in maintenance of the choriocapillaris [48]. Taken together, concerns about the consequences of global inhibition of VEGF signaling were and remain significant. This is corroborated by serious adverse events associated with systemic Avastin treatment, which include black box warnings for gastrointestinal perforations, wound healing complications, and hemorrhage. Warnings and precautions also include myocardial infarction, stroke, and hypertension [49]. It was with such considerations in mind and with the intent of striking the optimal balance between efficacy and safety that we chose VEGF-165 as a target for therapeutic intervention. This decision, as we will see below, had a profound impact on the clinical success of Macugen. When we were ready to begin our first SELEX experiments in 1993, VEGF was not available commercially. Fortunately, NeXstar (then NeXagen) had entered into a licensing agreement with Genentech (Genentech was one of the original investors) and this allowed us to obtain VEGF-165 as a generous gift from Dr. Napoleone Ferrara [10].

Our initial goal was to develop VEGF aptamers for the treatment of malignancies. At the same time, the importance of VEGF in the progression of ocular disorders such as diabetic retinopathy and macular degeneration was becoming clear based on animal models of ocular neovascularization as well as elevated ocular VEGF levels in patients with neovascular lesions [50–52]. In the eye, VEGF is secreted by retinal pigmented epithelial (RPE) cells from the basal side, directing paracrine stimulation toward choroidal blood vessels, which express high levels of VEGFRs [53]. In exudative (wet) age-related macular degeneration (AMD), new blood vessels that originate from the choroid break through Bruch's membrane to disrupt the RPE cells. The vascular leakage and hemorrhage from these nascent vessels lead to loss of central vision. Overall, broad evidence was mounting to support the conclusion that VEGF is the angiogenic factor X hypothesized by Michelson in 1948 to be produced by the ischemic retina [54].

Although ophthalmology was not our initial focus, there were a number of advantages inherent to ocular disorders that were difficult to ignore. VEGF was clearly a key driver of pathological angiogenesis in the eye and, unlike in cancer, all cell types involved were genetically stable—that is, they were not likely to mutate away from VEGF dependence. The VEGF aptamer could be delivered locally in small amounts, thus reducing considerable concerns related to cost of goods. The ocular pharmacokinetic profile of the aptamer was very good (see next section), suggesting that infrequent dosing was possible. This was not the case with half-life in plasma, where antibodies hold a considerable advantage. While high concentration could be reached in the eye, systemic exposure was minimal, thus reducing concerns related to inhibiting VEGF throughout the body. Most importantly, AMD represented a huge unmet medical need with increasing incidence as the population aged.

## SELEX with VEGF-165 and the Composition of NX1838

SELEX experiments targeting VEGF-165 represent a good illustration of the evolution in our thinking about best nucleic acid libraries for therapeutics. We began with unmodified RNA and demonstrated that VEGF-165 was an amenable target for SELEX [9]. Other than a proof of principle that inhibitory aptamers could be identified, this was a dead end because even a single unprotected ribopyrimidine would render an aptamer labile to RNAse.

We next tried a 2′-aminopyrimidine library and again identified several families of aptamers with high affinity and potent inhibitory activity [5]. As expected, the inherent nuclease resistance of aptamers derived from a 2′-aminopyrimidine library was vastly superior to that of RNA aptamers [5,55,56]. We further improved nuclease resistance by substituting most of the ribopurines with 2′-methoxy (2′-OMe) purines [57]. However, 2′-aminopyrimidine-based libraries had two shortcomings. First, the 2′-amino group needed to be protected during solid-phase synthesis in a manner that reduced the coupling efficiency, driving up the manufacturing cost. Second, 2′-amino substitutions were known to reduce the stability of duplexes compared with unmodified RNA or DNA, which could limit the binding affinity based on entropic considerations.

**FIG. 2.**
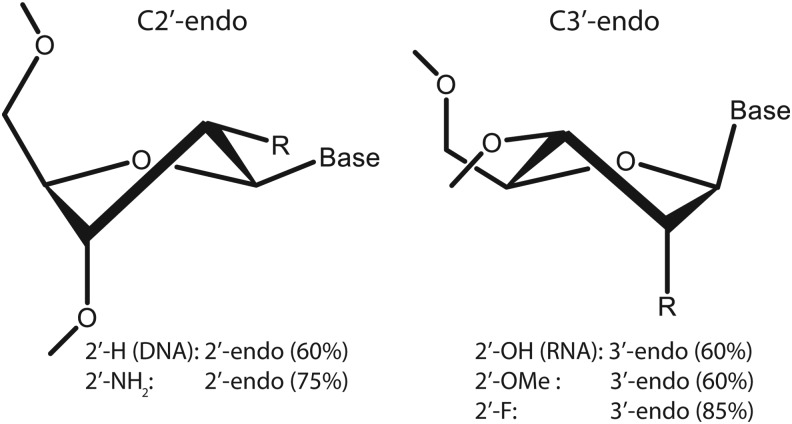
Sugar pucker preferences as a function of 2′-position substitution (adapted from Guschlbauer and Jankowski [131]).

We therefore considered 2′-fluoropyrimidine RNA libraries, from which we identified another set of inhibitory aptamers that as a group had the highest affinity for VEGF-165 [58]. It was from this set of SELEX experiments that Macugen was identified. This library solved both concerns related to the 2′-amino RNA libraries. The 2′-F group did not require protection, and the duplex stability was enhanced compared with unmodified RNA. In addition, the conformational preference for the C3′-endo conformation of 2′-F ribose was even greater than that of 2′-OH ribose and comparable with 2′-OMe (Fig. 2). This is likely related to the observation that most ribopurines could be substituted with 2′-OMe groups. For example, in clone t44, all but two purines tolerated 2′-OMe substitution (t44-OMe). To attenuate 3′-5′ exonucleases, we added a 3′-3′ inverted deoxythymidine (idT) cap to all aptamers designated for *in vivo* testing [59].

From the three families of aptamers identified in this set of SELEX experiments, we selected t44-OMe for further development primarily on the basis of its relative inhibitory potency in receptor binding and vascular permeability assays. Another reason for selecting t44-OMe was the fact that it retained the highest binding affinity to the VEGF/placenta growth factor (PlGF) heterodimer, which had been isolated from tumor cell line supernatants [60,61]. Since we did not observe any binding to the PlGF homodimer or to the reduced monomeric form of VEGF-165, this meant that t44-OMe retained some binding affinity for the native VEGF monomer in the context of a dimer [58].

**FIG. 3.**
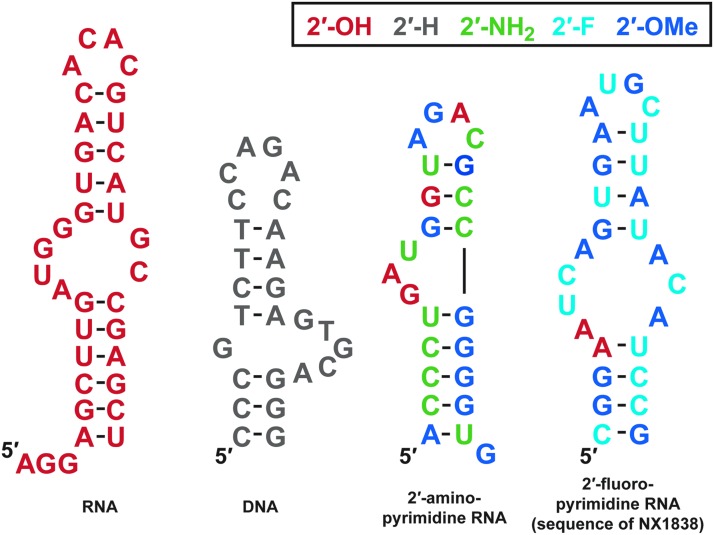
Sequences and predicted secondary structures of aptamers derived from systematic evolution of ligands by exponential enrichment (SELEX) experiments with different nucleic acid starting libraries. In this and subsequent figures the idT cap is not shown.

**FIG. 4.**
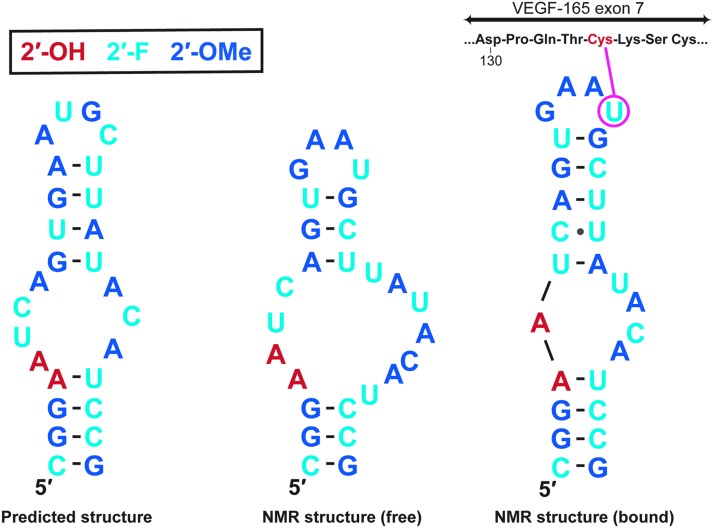
Predicted secondary structure and free and bound NMR structures of t44-OMe. Photo-cross-linking site between the aptamer and Cys-134 on VEGF-165 is indicated.

To complete the screening of nucleic acid libraries available to us at the time, we also identified a set of single-stranded DNA aptamers to VEGF-165 [62]. The composition and the predicted secondary structures of aptamers derived from RNA, DNA, 2′-aminopyrimidine, and 2′-fluoropyrimidine libraries are shown in Fig. 3. It is worth noting that these sequences are different from one another, showing that the composition of nucleic acid libraries greatly influences the resulting sequence solutions to high-affinity binding [63]. All of the aptamers were specific for VEGF-165 and showed no binding to VEGF-121. Photo-cross-linking experiments with the 2′-fluoropyrimidine aptamers helped us establish that the aptamers were binding to the exon 7-encoded domain of VEGF-165 (Fig. 4) [58]. Nuclear magnetic resonance (NMR) spectroscopy studies with t44-OMe performed by Lee *et al*. confirmed this observation and showed that the aptamer (which had not only many elements of the predicted secondary structure but also some differences) undergoes an induced fit with further tightening upon binding to VEGF (Fig. 4) [64,65].

Aside from nuclease resistance, the clearance of nucleic acids is limited by their size, and with the molecular mass of about 10 kDa, t44-OMe is readily filtered by the kidneys. To improve the pharmacokinetic properties of t44-OMe for *in vivo* testing, we explored adding a number of functional groups, including cholesterol [66], polyethylene glycol (PEG) [67,68], diacylglycerol, and liposome-embedded aptamers [69]. Aside from the advantage of being used in other drugs, PEG also improved the inhibitory activity of t44-OMe in the vascular permeability assay even though its binding affinity was reduced compared with the unconjugated aptamer by about fourfold [58].

*In vivo*, the addition of PEG improved the plasma half-life of t44-OMe in rats following intravenous (IV) administration. Improvement was dependent on the molecular weight of the PEG, with 20 and 40 kDa conjugates exhibiting half-lives of 3.2 and 6 h, respectively, compared with 0.3 h for unconjugated t44-OMe (Table 1) [70]. In primates, the plasma half-lives of the 40 kDa PEG conjugate were 9.3 and 12 h following IV and subcutaneous administration, respectively [71]. In the eye, following intravitreal (IVT) injection, the half-lives of this same 40 kDa PEG conjugate were much longer: 90–99 h in rhesus monkeys, 83 h in New Zealand White (NZW) rabbits, and 111 h in Dutch-belted (DB) rabbits (Table 2) [70,72,73].

**Table 1.** Plasma Terminal Half-Lives (Hours) Following a Single Intravenous Bolus Dose of Unconjugated (No PEG) t44-OMe (with a 5′-Amine) or As Conjugated to a Linear 20 kDa PEG or As Conjugated to a Branched 40 kDa PEG (Macugen)

	*No PEG*	*20 kDa PEG*	*40 kDa PEG (Macugen)*				
*Dose (mg/kg)*	*1.0*	*1.0*	*0.005*	*0.05*	*0.10*	*1.0*	*10*
SD rat [70]	0.30	3.2				6.0	
CD-1 mouse [70]					4.0	3.5	4.5
Beagle dog [70]			2.0	1.8			
Rhesus monkey [71]						9.3	

OMe, methoxy functional group; PEG, polyethylene glycol; SD, Sprague-Dawley.

**Table 2.** Macugen Vitreous Humor Terminal Half-Lives (Hours) Following a Single Intravitreal Bolus Dose

	*Dose (mg/eye)*		
*Species*	*0.5*	*1.0*	*2.0*
Rhesus monkey [72]	98.7	94.1	89.9
DB rabbit [70]	111	—	—
NZW rabbit [73]	83.0	—	—

DB, Dutch belted; NZW, New Zealand white.

## From NX1838 to Macugen

The PEGylated form of t44-OMe was renamed NX1838, which was tested in a battery of preclinical tests required for an investigational new drug (IND) application. This included demonstration that in primary endothelial cells, NX1838 inhibited the binding of VEGF to its receptors as well as VEGF-induced receptor phosphorylation, calcium influx, and cell proliferation [74]. *In vivo*, NX1838 inhibited VEGF-induced vascular permeability, retinopathy of prematurity (ROP), and tumor growth [73]. The latter two efficacy models are especially relevant because they both rely on endogenously produced VEGF, in which various isoforms are represented in natural proportion.

In the ROP model, retinal neovascularization is induced in neonatal mice simply by controlling their exposure to oxygen. Following a period of hyperoxia, mice are returned to normal room air. The angiogenic response that ensues is known to be dependent on VEGF induced by the change in oxygen levels. This condition is essentially identical to that observed in premature infants who often need to be temporarily placed in hyperoxic chambers because their lungs are not fully developed. In this model, Macugen delivered systemically by intraperitoneal injection inhibited up to 80% of retinal neovascularization. In the tumor xenograft experiments, the growth of human tumors such as A673 rhabdomyosarcoma was inhibited by 71%–76% [73]. Further corroborating the essential role of VEGF-165 in pathological angiogenesis in the eye, Ishida *et al*. showed that the baseline ratio of VEGF-165/VEGF-121 (VEGF-164/VEGF-120 in rodents) increased more than 10-fold (to about 25) during ischemia-induced pathological angiogenesis [75].

Nonclinical safety studies were performed following IVT or IV administration (Table 3) [70,76]. For IVT administration, single-dose toxicology studies were conducted in NZW rabbits and rhesus monkeys and repeated-dose studies were conducted in DB and NZW rabbits, rhesus monkeys, and beagle dogs. Macugen was well tolerated and no significant toxicological findings were made in any study even when administered to beagle dogs at 3.0 mg/eye every 2 weeks for 9 months (20 doses) or to NZW rabbits at 2 mg/eye every 2 weeks for 6 months [77].

**Table 3.** List of Nonclinical Toxicology Studies for Macugen [70,76]

*Study type*	*Test*	*Species*	*Dose/schedule/route*
Gene toxicity	Mouse lymphoma assay	Mouse L5178Y TK^+/−^	Up to 5 mg/mL
	Ames tests	*Salmonella-Escherichia coli*	Macugen or 2′-F cytidine or 2′-F uridine or 2′-OMe guanosine or 2′-OMe adenosine up to 5 mg/mL
	Cell transformation assays	Syrian hamster embryo cells	Macugen up to 1 mg/mL or 2′-F cytidine up to 3.5 mg/mL or 2′-F uridine up to 3.5 mg/mL, 7-day exposure
	Chromosome aberration assays	Human lymphocytes	2′-F cytidine or 2′-F uridine or 2′-OMe guanosine or 2′-OMe adenosine up to 5 mg/mL
	Micronucleus assay	CD-1 mouse	1, 10, or 100 mg/kg, IV
Safety pharmacology	Neurological effects	SD rat	0.007, 0.020, or 0.065 mg/kg, IV
	Respiratory system	SD rat	0.007, 0.020, or 0.065 mg/kg, IV
	Cardiopulmonary system [77]	Beagle dog	Loading dose 0.0045, 0.0135, or 0.045 mg/kg, IV maintenance dose, 0.002, 0.006, or 0.020 mg/kg/h, IV
Single-dose toxicity	Acute ocular toxicity	NZW rabbit	PBS in one eye, 0.5 mg/eye in other eye, IVT
	Acute ocular toxicity [72]	Rhesus monkey	0, 0.25, 0.5, 1, 1.5, or 2 mg/eye, bilateral IVT; some monkeys received multiple doses after 29-day washout
	Acute ocular toxicity	Rhesus monkey	0.5 mg/eye, IVT
	Acute systemic toxicity	Rhesus monkey	0, 5 mg/kg, IV by 1-h infusion
	Acute systemic toxicity	SD rat	0, 50, 150, or 450 mg/kg, IV
Repeated-dose toxicity	11-week ocular toxicity	DB rabbit	0 mg/eye six doses every 2 weeks; 0.1 mg/eye four doses every 2 weeks, then 2 mg/eye on weeks 9 and 11, IVT; 0.3 or 1.0 mg/eye weeks 1, 3, 5, 7, 9, and 11, IVT
	3-month systemic toxicity	SD rat	0, 0.1, 1, or 10 mg/kg daily for 13 weeks, IV
	3-month ocular toxicity	Rhesus monkey	6 semimonthly 0, 0.1, 0.25, and 0.5 mg/eye or 4 semimonthly 0.1 mg/eye, followed by 2 semimonthly 1 mg/eye, IVT
	3-week ocular toxicity	Beagle dog	0 or 2 mg/eye weekly for 3 weeks, IVT
	6-month ocular toxicity [77]	NZW rabbit	Bilateral 0, 0.2, 0.67, or 2 mg/eye every 2 weeks for 6 months IVT
	9-month ocular toxicity [77]	Beagle dog	0, 0.3, 1, or 3 mg/eye every 2 weeks for 9 months, IVT
	Toxicology of 2′-F pyrimidine nucleotides [187]	Fischer 344 rat	2′-F cytidine or 2′-F uridine 0, 5, 50, or 500 mg/kg daily for 90 days, IV
	Toxicology of 2′-F pyrimidine nucleotides [187]	Woodchuck	2′-F cytidine or 2′-F uridine 0, 0.75, or 7.5 mg/kg daily for 90 days, IV
Reproductive toxicity	Developmental toxicity	NZW rabbit	0.067, 0.2, 0.67, or 2 mg/eye gestation days 6, 13, and 19, IVT
	Developmental toxicity	CD-1 mouse	1.0, 6.5, or 40 mg/kg gestation days 6–15 or 6–17, IV
Special toxicity	2′-F pyrimidine incorporation into nucleic acid	Rat	10 mg/kg daily for 90 days, IV
	2′-F pyrimidine incorporation into nucleic acid [188]	Rat and woodchuck	2′-F cytidine or 2′-F uridine; rat 5, 50, 500 mg/kg daily for 90 days, IV; Woodchuck 0.75 and 7.5 mg/kg daily for 90 days, IV
	Lymphocyte stimulation assay	Human and mouse lymphocytes	*In vitro*, up to 40 μM
	Immunogenicity testing	Balb/c mouse	Mouse up to 100 μg weekly for 8 weeks, IV
		SD rat	Rat up to 10 mg/kg daily for 3 months, IV
		DB rabbit	Rabbit up to 1 mg weeks 1, 3, 5, 7, 9, and 11, IVT

Doses are for Macugen unless otherwise indicated.

2′-F, 2′-fluoro; IVT, intravitreal; PBS, phosphate-buffered saline; TK, thymidine kinase.

Macugen was also well tolerated following single and repeated IV-dose studies in Sprague-Dawley rats and rhesus monkeys. Systemic effects were only observed in rats in the 13-week IV study. Findings included vacuolated cells in multiple organs, which may have contained PEG [78]. Safety pharmacology studies in dogs [77] and rats found no effects on the cardiopulmonary system or neurological deficits.

No immune responses to Macugen were observed. No developmental abnormalities were observed in reproductive toxicity studies in rabbits and mice. Metabolism studies performed *in vitro* and *in vivo* showed that Macugen was still susceptible to nuclease digestion. This was initially a cause for concern due to the potential for incorporation of modified nucleosides into cellular DNA and RNA. However, such fears were allayed when *in vitro* testing showed that the genotoxic potential of full-length Macugen and the modified nucleosides themselves was low [76].

In January of 1999, clinical testing of Macugen sponsored by NeXstar Pharmaceuticals began with an open-label, dose-escalating safety study conducted in 15 AMD patients with choroidal neovascularization (CNV) (wet AMD) who received a single administration of 0.25, 0.5, 1, 2, or 3 mg Macugen/eye [73]. No dose-limiting toxicity was observed, but the viscosity of the formulation prevented any further increase in dose. At 3 months, 12 of the 15 patients showed stable or improved vision with 4 patients showing an increase in best corrected visual acuity (BCVA) of 15 letters (three lines) or more as assessed on the Early Treatment of Diabetic Retinopathy Study (ETDRS) chart.

Encouraged by this phase 1 result, an open-label, multidose safety study was undertaken, now by the newly formed company Eyetech Pharmaceuticals, which licensed the Macugen program following the acquisition of NeXstar by Gilead Sciences [79]. Patients with subfoveal CNV secondary to AMD received three doses of Macugen (3 mg/eye) at 28-day intervals with or without photodynamic therapy (PDT). PDT was given to patients with predominantly classic CNV 5–10 days before the first Macugen dose.

No drug-related serious adverse events were noted in the 21 patients who received Macugen, and 18 patients completed the 3-month treatment period. Of the eight patients who received Macugen alone and completed the study, seven had stable or improved vision at 3 months. Two of these patients had a three-line or greater improvement on the ETDRS chart. Of the 10 patients who received cotherapy and completed the study, 9 had stable or improved vision with 6 showing a three-line or greater improvement. Although a conclusion regarding efficacy could not be drawn from this small and uncontrolled study, the results did compare favorably with historical controls, especially with regard to BCVA improvement, and were compelling enough to enter into prospective randomized, controlled clinical trials.

Macugen was approved on the basis of two controlled, double-masked, and identically designed studies in patients with neovascular AMD [80]. Approximately 1,200 patients were randomized to receive control (sham treatment) or 0.3, 1, or 3 mg Macugen administered by IVT injection every 6 weeks for 48 weeks. The two trials enrolled patients with classic, minimally classic, occult, and mixed lesions (essentially all-comers) with a total lesion size up to 12 optic disc areas and baseline visual acuity in the study eye between 20/40 and 20/320 (referred to as VISION studies). The primary efficacy end point was the proportion of patients losing less than 15 letters of visual acuity (defined as vision maintenance) from baseline to the 54-week assessment.

Macugen-treated groups exhibited a statistically significant benefit in both trials. In the first study, 73% of patients receiving 0.3 mg Macugen maintained visual acuity compared with 60% in the sham injection group. A similar outcome was observed in the second study: 0.3 mg Macugen 67% versus sham injection 53%. The outcome at the 54-week assessment was comparable across the three Macugen dose groups, with no evidence of enhanced benefit in the higher dose groups [80]. On average, patients treated with 0.3 mg Macugen as well as sham-treated patients continued to experience vision loss. However, the rate of vision decline was slower in the Macugen-treated group than in the sham-treated group.

**FIG. 5.**
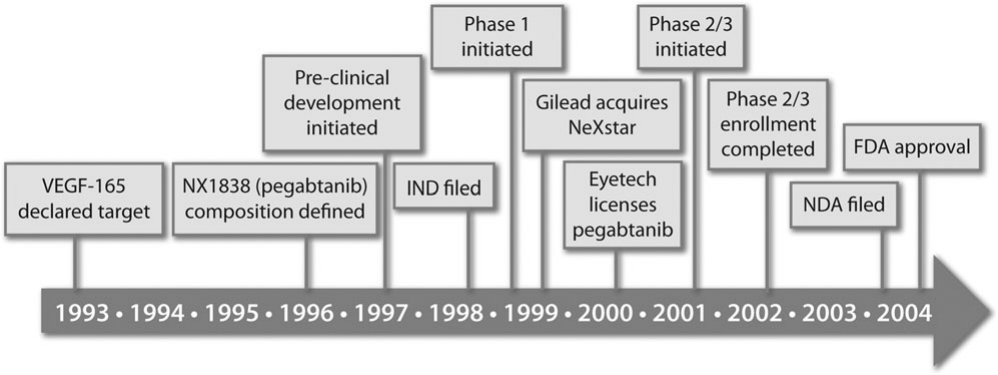
Time line for the development of Macugen.

Vision gain defined as an improvement in BCVA of three lines or greater was observed in 6% of patients in the 0.3 mg Macugen group compared with 2% of sham injection controls. Consistent with preclinical observations, no antibodies to Macugen were detected in any of the patients in the registration studies [80]. Macugen received FDA approval in December of 2004 for the treatment of wet AMD at the dose of 0.3 mg, given as an IVT injection every 6 weeks, 8 years after NX1838 was nominated as a development candidate (Fig. 5).

At the end of the first year, ∼1,050 of the original 1,200 patients were rerandomized to either continue the same treatment through week 102 or to discontinue treatment. The benefit of Macugen continued. Patients rerandomized to continue 0.3 mg Macugen demonstrated a 45% relative benefit in mean change in vision at the end of 102 weeks compared with those receiving usual care [81].

## Nonselective VEGF Antagonists

As the first anti-VEGF therapy to receive FDA approval for ophthalmic use, as well as the first aptamer therapeutic, Macugen reached peak sales of $185 M (United States) in its first full year of sales (2005). Since that time, it has been substantially displaced by protein-based anti-VEGF therapeutics: Lucentis (ranibizumab), Avastin, and Eylea (aflibercept). These agents produce a considerably greater BCVA improvement than Macugen. For example, compared with a three-line gain in 6% of the patients treated with Macugen for a year [80], about one-third of patients experienced such improvement with Lucentis [82,83], Eylea [84], and Avastin [85]. The fact that Macugen was dosed every 6 weeks compared with every 4 weeks for Lucentis is unlikely to be the reason for the difference in efficacy since a 10-fold higher dose of Macugen (3 mg per eye) exhibited similar efficacy to the label dose. The most likely explanation is the fact that all three of the protein-based drugs inhibit all isoforms of VEGF, including VEGF-121 and VEGF-110, whereas Macugen is selective for VEGF-165 [86–88].

When sight is involved, the immediate effect of improving vision is favored over potential safety concerns related to long-term inhibition of VEGF. Nevertheless, such safety concerns do remain. It is clear that VEGF plays a maintenance role in the eye, for example, for retinal neurons [47], and that ongoing and complete inhibition of this activity may be counterproductive in the long term [89]. In this context, the observation that VEGF-120 protected the retina against hypoxic injury with equal effectiveness to VEGF-164 (mouse homologs of VEGF-121 and VEGF-165) is potentially important [47].

In addition, persistent inhibition of VEGF may undermine the ability of choroidal vasculature to nourish the retina [89]. For these reasons, selective inhibition of VEGF-165 has been proposed as a treatment option in an induction–maintenance regimen, where the treatment is initiated with a nonselective VEGF antagonist, followed by a switch to Macugen, as a way of finding the best balance between efficacy and safety [90–92]. Whether such an alternative use of Macugen ever takes hold in clinical practice remains to be seen, but the option for such selective treatment does exist. Of course some side effects have been observed with Macugen, including endophthalmitis, increases in intraocular pressure, and rare cases of anaphylaxis/anaphylactoid reactions, including angioedema [93–96].

## Beyond VEGF Inhibition

Anti-VEGF therapy has transformed the treatment of wet AMD (and also other VEGF-dependent conditions such as diabetic retinopathy, macular edema, and retinal vein occlusion) [97]. Nevertheless, not all AMD patients respond to such therapy, and in those who do, the response can be transient or incomplete. Even with nonselective antagonists, only about one-third of patients respond with a BCVA gain of three lines or more [82–85]. Concurrent with our early-stage development of Macugen, it was becoming clear that new blood vessels become less susceptible to VEGF withdrawal as they mature and that concurrent inhibition of VEGF and another growth factor, platelet-derived growth factor (PDGF), may result in regression of such partially matured blood vessels. In the following section, we recount the events that led to the identification of Fovista.

The initial growth of a new blood vessel is a highly dynamic process characterized by considerable plasticity during sprouting and pruning of the new vessels as they are remodeled into final vascular architecture [98]. Initially comprised of endothelial cells, newly formed immature blood vessels require VEGF for survival and are highly susceptible to VEGF inhibition. VEGF prevents apoptosis in endothelial cells during periods of serum starvation by inducing antiapoptosis proteins [28,99].

As new vessels mature, the association of endothelial cells with periendothelial cells or pericytes leads to gradual loss of VEGF dependence as the remodeling of the vascular network ceases [98,100]. Pericytes are contractile cells of myeloid origin (similar to smooth muscle cells) that encircle and stabilize microvessels in various tissues and organs. In tumor xenograft models, selective regression of immature vessels occurs following VEGF downregulation, where endothelial cells undergoing apoptosis are predominantly confined to vessels lacking pericytes [100].

## PDGF B-Chain and AMD

The molecular mechanism underlying the recruitment of pericytes to new blood vessels has been substantially elucidated over the past two decades. It is now well established that PDGF plays a central role in pericyte recruitment and microvessel maturation. PDGF is a ubiquitous mitogen and chemotactic factor for many connective tissue cells, that occurs as disulfide-linked dimers comprised of two homologous chains, A and B [101]. The biological function of all PDGF forms is mediated through binding to two cell surface proteins, PDGF receptors α and β [102,103].

PDGF binding induces receptor dimerization, which leads to autophosphorylation at specific intracellular tyrosine residues [104]. Since the A-chain binds only to the α-receptor and the B-chain to both receptors, the α-receptor homodimer binds all three PDGF isoforms and the αβ heterodimer binds PDGF-AB and PDGF-BB, while the β-receptor homodimer binds only PDGF-BB with high affinity [102–104]. Thus, the differences in cellular responses induced by these three PDGF dimers are governed by their receptor-binding properties and the profile of receptor expression on target cells.

Other PDGF variants have also been described more recently, such as PDGF-CC, which binds specifically to the PDGF α-receptor in the context of both αα- and αβ-receptor dimers (thus resembling PDGF-AB in activity), and PDGF-DD, which binds specifically the β-receptor and exclusively interacts with the ββ-receptor homodimer [105–107].

The role and relative contribution of PDGF-C and PDGF-D, which (unlike PDGF-A and PDGF-B) only exist as homodimers, are incompletely understood. What is clear is that some redundancy and the potential for fine-tuning exist in signaling through the PDGF receptors, with PDGF B-chain being the only ligand capable of activating both receptors. PDGF-D appears to require proteolytic cleavage for full activity; however, there are no proteases secreted by cells and tissues in the eye capable of cleaving full-length PDGF-DD, so PDGF-B may be the only fully active ligand for the β-receptor at this site [107,108].

During new blood vessel maturation, PDGF-BB secreted by endothelial cells acts in a paracrine manner to recruit pericytes expressing PDGF β-receptors [109,110]. Since endothelial cells express PDGF β-receptors, PDGF-B is also a direct mitogen and chemotactic factor for endothelial cells [111]. PDGF B-chain and PDGF β-receptor knockout mice, which have a similar phenotype and die just before birth, have abnormally dilated blood vessels and develop numerous microaneurysms [112,113]. This phenotype arises, in part, because sprouting endothelial cells are unable to recruit PDGF β-receptor-positive pericyte progenitor cells, leading to mechanical instability of blood vessels [114]. Conditional knockout mice, in which targeted deletion of the PDGF-B gene is restricted to endothelial cells, also exhibit impaired recruitment of pericytes to blood vessels. Although these mice survive to adulthood, they exhibit some of the same microvessel and organ abnormalities as PDGF B-chain and PDGF β-receptor knockout mice [115].

It is relevant to note that VEGF also enhances the coating of retinal endothelial cells with pericytes in neonatal rats [98]. Pericytes *in vitro* express VEGFR1 (but not VEGFR2) and its expression is upregulated under hypoxic conditions [116,117]. As with monocytes that also express VEGFR1, VEGF has been shown to stimulate pericyte migration in a dose-dependent manner [118]. Last, pericytes are a known source of VEGF, suggesting that blood vessel maturation involves a combination of autocrine and paracrine signals facilitated by tight physical association of these two cell types [118,119].

Despite some redundancy of signaling between endothelial cells and pericytes, notably by the angiopoietin–Tie receptor system [110,120–122] (in fact, an aptamer inhibitor of angiopoietin-2 has been shown to be an antiangiogenic agent [123]), simultaneous inhibition of PDGF-B and VEGF pathways is sufficient to cause new blood vessel regression.

In a mouse model of spontaneous pancreatic islet cancer, Bergers *et al*. have shown that a combination of VEGF-selective and PDGF-selective tyrosine kinase inhibitors was more effective in attenuating tumor-associated angiogenesis and tumor burden compared with the effects observed with either agent alone [124]. The Simian virus 40 (SV40) oncogene-expressing RIP1-Tag2 tumor cells in this model do not express any of the PDGF ligands or receptors and so the effects of the combined treatment can be attributed to those related to tumor-associated vasculature [124]. Erber *et al*. have obtained similar results with the same pair of tyrosine kinase inhibitors in a rat C6 glioma subcutaneous tumor xenograft model [120].

In both studies, the combined inhibition of both VEGF and PDGF signaling pathways was required to achieve regression of established tumor blood vessels by causing disruption of the association between endothelial cells and pericytes and the consequent increase in susceptibility to proapoptotic effects associated with VEGF inhibition. Importantly, disruption of the existing vasculature in normal mouse tissues was not observed with this systemic combination treatment for reasons that remain to be fully elucidated [120,124]. Pietras and Hanahan showed that dual inhibition of VEGF and PDGF with tyrosine kinase inhibitors was synergistic with cyclophosphamide chemotherapy in the RIP1-Tag2 transgenic mouse model of pancreatic cancer [125].

These observations have recently been corroborated with antibody-based inhibitors, which are generally much more specific antagonists than the small-molecule tyrosine kinase inhibitors known to act on a broad range of kinases, thus complicating the interpretation of the results. Treatment with the combination of inhibitory antibodies to PDGF β-receptor and VEGFR2 resulted in enhanced antitumor and antiangiogenic effects in mouse xenograft models of nonsmall cell lung cancer and pancreatic cancer [126].

In another recent study, a single-chain bispecific antibody fragment variable (Fv) fragment fused to both termini of a human fragment crystallizable (Fc) domain (scFv-Fc-scFv, a tetrameric construct), targeting human PDGF β-receptor and VEGF, inhibited pericyte–endothelial cell association and vessel sprouting *in vitro* more completely than Avastin alone [127]. Because this construct does not cross-react with mouse PDGF β-receptor and VEGF, its efficacy could not be adequately tested in mouse *in vivo* models. Nevertheless, tumor growth of A673 rhabdomyosarcoma xenografts, which are known to produce a large amount of human VEGF, was inhibited to a degree comparable with that observed with bevacizumab [127].

In ocular angiogenesis, Jo *et al*. have examined the effects of combined inhibition of VEGF and PDGF-B signaling in mouse models of corneal neovascularization (CorNV) and CNV using Macugen and an antimurine PDGF β-receptor antibody [128]. The CNV model, induced by laser burn of the retina, is relevant because it mimics human AMD. The CorNV model, induced by limited corneal injury, is useful because new vessels are clearly visible in a typically avascular cornea and because corneal vessels do not naturally regress. In both models, combined blockade of VEGF and PDGF-B signaling pathways was superior to monotherapy in both prevention and treatment modalities. In the CorNV model, combination treatment resulted in regression of established vessels [128].

## SELEX with PDGF B-Chain and the Composition of NX1975

Fovista was identified from the SELEX experiment initiated with a single-stranded DNA library in the spring of 1995. At that time, this was more of an experimental selection since there was a bias against single-stranded DNA libraries, which were believed to be less conformationally diverse compared with single-stranded RNA libraries. However, DNA had the appeal of being much more stable than unmodified RNA and, more importantly, it was easier to synthesize than RNA.

We chose PDGF-AB as the SELEX target with the expectation that we would get a range of ligands: some targeting the A-chain, some the B-chain, and some the interface between the two chains unique to the heterodimer. Instead, all of the clones were specific for the PDGF B-chain, a surprising observation that showed us that ligands targeting a dominant epitope on a protein can outcompete other ligands in an affinity-enriched pool (PDGF A-chain aptamers would have to be identified with PDGF-AA).

Most of the sequences in the affinity-enriched pool could form a common consensus motif: a three-way helix junction with a three-nucleotide loop at the branch point. This motif was supported by base-pairing covariation in the three stems (which, in the absence of structures, is one of the most compelling pieces of evidence to support the existence of secondary structure of aptamers) as well as truncation experiments. However, for the motif to become obvious, correct alignment of the sequences was essential and, in this case, difficult because the conserved motif is the core of the junction, with less conserved regions on the periphery. Therefore, short, circularly permuted, and discontiguous conserved sequence segments were separated by regions of variable sequence and length [129]. We now know that such primary structures often define consensus motifs of aptamers.

**FIG. 6.**
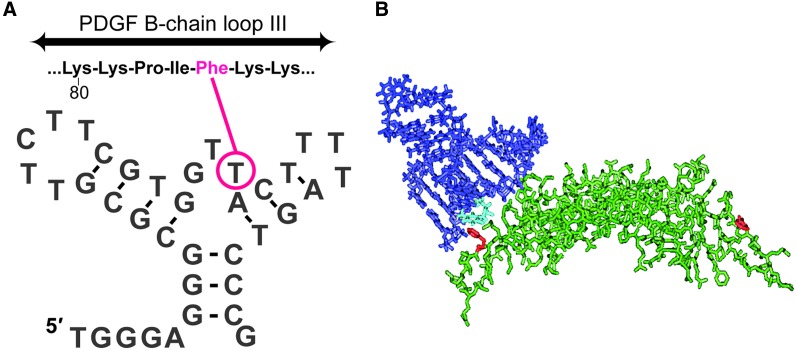
**(A)** Photo-cross-linking of aptamer 20t to Phe-84 on platelet-derived growth factor (PDGF) B-chain. **(B)** Model of the interaction based on the crystal structure of PDGF-BB and a superimposed model of the aptamer based on the cross-linking contact.

Three representative truncated clones that conformed to this motif exhibited high-affinity binding to PDGF-AB and PDGF-BB (93–147 pM) and much lower affinity for PDGF-AA (47–72 nM). Upon reduction of disulfide bonds in PDGF-AB, high-affinity binding is lost [129]. Photo-cross-linking studies with PDGF-AB showed that an unpaired nucleotide at the helix junction is in close contact with phenylalanine 84 near loop III of PDGF B-chain (Fig. 6).

Together, these observations confirmed that the aptamers bound to the B-chain of PDGF in the context of the native homo- or heterodimer at a site that overlapped with the receptor-binding region [129]. All three truncated clones inhibited the binding of PDGF-BB, but not PDGF-AA, to PDGF α-receptors on porcine aortic endothelial cells and inhibited PDGF-BB-induced DNA synthesis in porcine aortic endothelial cells expressing the PDGF β-receptor.

**FIG. 7.**
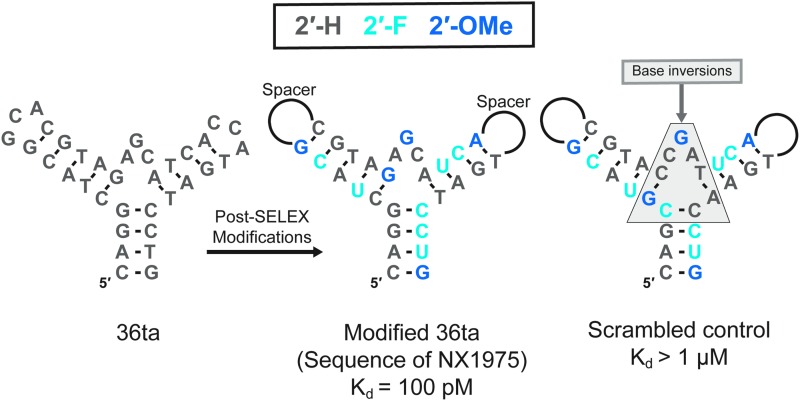
Sequence and predicted secondary structure of PDGF B-chain aptamer 36ta (36t with two base pairs deleted from stem 1) and post-SELEX substitutions leading to the nucleic acid component of NX1975. Inverting the bases in the helix junction without disrupting the predicted secondary structure domain eliminates high-affinity binding.

We selected clone 36t for further studies, taking into consideration binding affinity, *in vitro* potency, and its relatively compact size. From its original truncation to a 39-mer, we were able to further shorten the molecule by replacing two trinucleotide loops with a pentaethylene glycol spacer and by deleting two additional base pairs at the base of stem 1. Knowledge of the structure allowed us to reduce the molecule to 29 nucleotides (Fig. 7) without compromising binding affinity [129,130].

To improve nuclease resistance, we synthesized a series of variants in which the deoxynucleotides were replaced with one or more 2′-F or 2′-OMe nucleotides. The outcome was a core sequence with 12 modified nucleotides (modified 36ta) that exhibited a 13-fold longer half-life in plasma compared with the unmodified parent DNA sequence. The lower tolerability to substitution at the 2′-position in this DNA aptamer (41%) compared with that achievable in RNA is likely a consequence of the preference of deoxyribose for the C2′-endo conformation compared with the preference for the C3′-endo conformation in ribose, 2′-F and 2′-OMe ribose (Fig. 2) [131]. The addition of 40 kDa PEG to this sequence resulted in NX1975, a construct with a terminal half-life in rat plasma of about 2 h [130].

## From NX1975 to Fovista

The activity of NX1975 was tested in several *in vivo* efficacy models. In a rat model of restenosis, NX1975 delivered by intraperitoneal injection twice daily for 2 weeks at doses ranging from 2 to 20 mg/kg/day, reduced the extent of intimal hyperplasia by 50% at the 2-week time point following balloon catheter injury to the carotid artery. The beneficial effect was accompanied by increased apoptosis of smooth muscle cells and possibly inhibition of their migration from the media to the intima of the vessel wall. All dose groups exhibited essentially the same effect (suggesting that EC_50_ was lower than 2 mg/kg/day), whereas the inverted sequence control was inactive.

This effect size was comparable with that observed with PDGF β-receptor antibodies and PDGF receptor kinase inhibitors [132]. However, reducing the treatment duration to 1 week after injury reduced the effect size at 2 weeks to levels below statistical significance. In addition, the benefit of treatment with NX1975 for 2 weeks was lost by 8 weeks, mostly because of ongoing matrix accumulation in the lesions, showing that either ongoing inhibition of PDGF B-chain or inhibition of another mediator of intimal hyperplasia is required for a sustained effect in this model [132].

In a rat model of complement-mediated kidney injury induced with anti-Thy 1.1 antibody, NX1975 delivered intravenously twice per day at a dose of 2.2 mg/kg/day for 7 days [130] or 5 mg/kg/day for 5 days [133] reduced mesangial cell proliferation, glomerular hypertrophy, podocyte damage, and glomerular macrophage influx by days 8–9. Importantly, this short-term, but potent, inhibition of PDGF B-chain resulted in a long-term protective effect in the kidney following acute injury. Through day 100 of the study, NX1975 reduced proteinuria, podocyte damage, glomerulosclerosis, tubulointerstitial damage, renal scarring, and matrix accumulation, essentially to levels observed in non-nephritic controls [133].

Importantly, NX1975 treatment reduced the expression of profibrotic cytokine transforming growth factor-β protein in the renal interstitium. The sequence-scrambled control exhibited none of the protective effects [130,133]. The more lasting effect of transient PDGF B-chain inhibition in this model compared with the reversible benefit observed in the restenosis model points to the greater importance of PDGF B-chain in glomerular injury compared with its role in large vessel injury. This is consistent with the observation that PDGF B-chain and PDGF β-receptor knockout mice do not have a mesangium, but do have major blood vessels [112,113].

In a rat colon carcinoma model in which PDGF β-receptor expression is confined to the tumor stroma, Pietras *et al*. showed that a 4-day treatment with NX1975 at a twice daily dose of 7 mg/kg/day reduced the interstitial pressure of established tumors by about 30% [134]. The effect was similar to that observed with a small-molecule tyrosine kinase inhibitor, STI571, which not only selectively inhibits PDGF β-receptor but also several other tyrosine kinases. Importantly, the reduction in interstitial tumor pressure was accompanied by an increase in perfusion of the tumor by a small-molecule tracer, indicating that selective inhibition of PDGF B-chain/PDGF β-receptor signaling within tumor stroma could increase the exposure of tumors to chemotherapeutic drugs [135,136]. In a subsequent study, Pietras *et al*. demonstrated that treatment with NX1975 or STI571 had no effect on the growth of tumors when used as monotherapy, but did enhance the antitumor effect of taxol and 5-fluorouracil [137].

In a fetal sheep model of pulmonary hypertension, which is accompanied with doubling of PDGF α- and PDGF β-receptor protein levels in the lung, Balasubramaniam *et al.* showed that twice daily infusions of NX1975 (2 or 4 mg/kg/day) directly into the left pulmonary artery for 7 days reduced by 47% the development of muscular thickening of the small pulmonary arteries associated with respiratory and terminal bronchioles [138]. In addition, right ventricular hypertrophy was decreased by 66% in the high-dose group. Together, these efficacy experiments confirmed the ability of NX1975 to potently and selectively inhibit PDGF B-chain-mediated effects *in vivo*.

NX1975 was renamed a few times as it made its way through several companies: ARC127, E10030, and ultimately Fovista. Most of the preclinical pharmacology studies have not been released; however, some of the pharmacokinetic and ocular efficacy studies have been published.

Akiyama *et al.* demonstrated a vitreous half-life of 98 h in DB rabbits [139]. Without PEG, the vitreous half-life was reduced to 43 h, as expected based on similar observations with Macugen. Akiyama also showed that both PEG and non-PEG forms, when injected into the vitreous cavity, could suppress extraretinal membrane formation and retinal detachment in a transgenic mouse model that overexpresses human PDGF-BB in photoreceptors. Untreated, these mice develop epiretinal membranes comprised of astrocytes, pericytes, and endothelial cells that lead to a tractional retinal detachment [140]. In eyes treated with a scrambled control aptamer, five of six eyes developed detachments. When treated with the non-PEG version, one of six eyes developed a partial detachment, and when treated with NX1975 (ARC127), no detachments were observed in any of the six eyes [139].

NX1975 as E10030 was taken into clinical testing by Ophthotech Corporation, which was founded by members of the same team that completed the development of Macugen (David Guyer and Samir Patel). In a phase 1 study, Fovista tested in combination with Lucentis resulted in a BCVA gain of three lines in 59% of patients at week 12 [141]. This is a considerably higher percentage compared with historical controls from clinical studies with Lucentis, in which about one-third of patients had a three-line improvement after 12 months of treatment [82,83]. In addition to improvement of vision, and for the first time in any AMD study, the combination treatment was accompanied with marked neovascular regression in all study participants with a mean decrease of 86% in the area of CNV.

Enhanced efficacy with combination treatment was recently corroborated in a phase 2 study of 449 patients with wet AMD. In this study, the patients were randomized to receive one of three treatment regimens administered every 4 weeks for 24 weeks: Fovista 0.3 mg in combination with Lucentis 0.5 mg; Fovista 1.5 mg in combination with Lucentis 0.5 mg; or sham in combination with Lucentis 0.5 mg.

The primary efficacy end point in the study was the mean change in BCVA from baseline at the week 24 visit. Patients receiving the combination of Fovista (1.5 mg) and Lucentis gained an average of 10.6 letters at 24 weeks compared with 6.5 letters for patients receiving Lucentis monotherapy (*P* = 0.019), representing a 62% additional benefit. Enhanced efficacy at the 1.5 mg dose compared with Lucentis monotherapy was demonstrated at every monthly time point with greater benefit at the 6-month time point than at the 3-month time point. Based on these results, Ophthotech is currently conducting four concurrent phase 3 studies [142].

## Complement and AMD

The complement system is a key component of innate and adaptive immune responses where it mediates events such as immune cell chemotaxis, phagocytosis, inflammation, and pathogen cell lysis [143]. Activation of complement occurs by one of three recognized pathways; the classical, lectin, and alternative pathways. The classical pathway can be activated not only by immunoglobulins such as IgM, or isotypes of IgG, but also by other mediators such as C-reactive protein. The lectin pathway is activated by the binding of a mannose-binding lectin to residues present on the outer surface of microorganisms, while the alternative pathway is activated by complex polysaccharides found on microbes or through autoactivation of complement component 3 (C3).

All three pathways involve serine proteases that exist as zymogens, each sequentially activated by proteolytic cleavage [144]. The pathways converge in the activation of C3, leading to the assembly of a complement component 5 (C5) convertase. Proteolysis of C5 by a C5 convertase results in a 9 kDa C5a fragment and a 200 kDa C5b fragment. C5a is the most potent anaphylatoxin of the complement system and mediates several biological activities, including immune cell chemotaxis, cytokine release, opsonization, production of oxidants, and increased vascular permeability. C5b is necessary for the assembly of the membrane attack complex (MAC), which can disrupt the lipid bilayer of cellular pathogens or damaged host cells, leading to cell lysis.

Both C5a and the MAC have been implicated in inflammatory-mediated tissue damage associated with human disease [145,146]. An inhibitor of proteolytic activation of C5 was considered a promising complement therapeutic since it could prevent an injury of this type while preserving upstream complement defense and clearance pathways [145,147,148].

As an aptamer inhibitor of C5 was being developed at NeXstar, a new hypothesis emerged for AMD pathogenesis. Inflammatory and immune-mediated events, including an overactivated or dysregulated complement system, were proposed [149,150]. Over the ensuing 15 years, several independent lines of evidence converged to support this view. Complement proteins were identified as components of drusen, the characteristic lesion of AMD. These included complement factor H (CFH), C3, and its split products, C5a, as well as the components of the MAC [149,151,152]. In addition, patients with AMD have altered plasma levels of complement factor D (CFD) and complement factor I (CFI) and elevated levels of the split products, Ba, C3a, and C5a [153–157].

Genetic studies showed an association between several genes and the development and/or progression of AMD (for review, Ref. [158]). The strongest known genetic risk factors are gene variants of CFH and age-related maculopathy susceptibility protein 2 (ARMS2). However, variants of the genes for complement factor B (CFB), CFI, C2, C3, and C9, also increase risk for this disease.

Based on this mounting evidence, the role of complement was tested in experimental animal models of AMD. Using a laser-induced CNV model in mice, Bora *et al.* showed that C3 and the MAC were deposited on neovascular complexes [159]. Animals that lacked functional C3 (C3−/−) or were depleted of complement by cobra venom factor did not develop CNV. In addition, these authors showed that a polyclonal anti-C6 antibody, which prevented MAC formation, also inhibited CNV. Nozaki *et al*. demonstrated that C3a and C5a could increase VEGF secretion by primary human RPE cells and that C3a and C5a receptor knockout mice had reduced VEGF expression in the RPE/choroid following laser-induced injury [151].

Antibody inhibitors of C3a and C5a, as well as a mirror-image mixed (l-)RNA/DNA aptamer inhibitor of C5a, have been shown to decrease laser-induced CNV in mice [151,160]. Using a spontaneous model of early-onset macular degeneration in cynomolgus monkeys, Chi *et al.* showed that compstatin, a cyclic peptide inhibitor of C3, was able to suppress and even reverse drusen formation when administered by weekly IVT injections for 6–9 months [161]. These studies suggest that inhibition of complement may be an effective clinical treatment for both wet and dry AMD and studies are now underway to test this idea in the clinic. Among these are not only several inhibitors of C5 (Zimura, eculizumab, LFG316) but also included are the anti-factor D antibody lampalizumab and the anti-C3 cyclic peptide POT-4, an analog of compstatin [146,162].

## SELEX with C5 and the Development of Zimura

Applying previous experience with modified nucleotide libraries, Greg Biesecker carried out SELEX experiments targeting C5 with the nuclease-resistant 2′-fluoropyrimidine RNA library [145]. His group identified a set of highly homologous aptamers that could inhibit the proteolytic activation of C5. Based on the sequence of a truncated inhibitory aptamer, a 38-mer named C5C6, a biased SELEX procedure [163] was used to discover aptamers with enhanced affinity for C5. This was essentially a new SELEX experiment, in which the random region was biased toward the sequence of C5C6 in an attempt to more fully explore the sequence space around this motif.

Following 8 rounds of biased SELEX, a C5-inhibitory aptamer was discovered that bound to C5 with 10-fold better affinity than C5C6 [145]. Using sequence information from both SELEX experiments, a new 38-mer sequence was selected that tolerated 2′-OMe substitution at all but three of the ribopurines. The addition of 3′-3′-linked deoxythymidine to the 3′ end and 40 kDa PEG to the 5′ end led to ARC1905 [164], which was subsequently named Zimura by Ophthotech.

In a nonrandomized, open-label phase 2a study in exudative AMD, 43 anti-VEGF therapy naive patients received 6 monthly doses of 0.3, 1, or 2 mg Zimura/ARC1905 in combination with 0.5 mg Lucentis [165]. At week 24, the mean increase in BCVA was 13.6, 11.7, and 15.3 letters for the low-, mid-, and high-dose groups, respectively, with a corresponding 46%, 47%, and 60% of patients gaining three or more lines. There were no dose-limiting toxicities. Similar to the Lucentis–Fovista combination, a considerably higher percentage of patients receiving the Lucentis–Zimura combination showed improved visual acuity compared with historical controls of patients receiving Lucentis monotherapy. Based on these positive results, Ophthotech has announced that they will continue trials for treatment of geographic atrophy.

## Vision for the Future: Conclusions and Perspective

Three aptamer-based drug candidates have reached clinical-stage testing in ocular indications and one has been approved. In addition, the antisense oligonucleotide, Vitravene/fomivirsen, was approved for cytomegalovirus retinitis in patients with aids [166]. The eye clearly represents a site where nucleic acid therapeutics in general [167,168] and aptamers in particular have distinct advantages over other classes of therapeutics. Considering these advantages is important for deriving broader lessons for indications that may represent the best opportunities for aptamer therapeutics.

With local delivery by IVT injection now routine, drugs can be introduced in high concentration at the site of pathology while minimizing systemic exposure, thus dramatically widening the therapeutic window. Clearance from the vitreous humor can occur through an anterior and a posterior route, which are both known to be dependent on molecular size [169,170]. The anterior route involves drug diffusion through the zonule fibers into the anterior chamber. The posterior route depends on permeation of the blood–retinal barrier.

In humans, the retinal exclusion limit—the maximum size of a macromolecule capable of freely diffusing across the retina—has been estimated to be ∼76 kDa, with similar values in other species [171]. However, this size barrier is apparently not absolute since Avastin was shown to be able to penetrate the rabbit retina at least to some degree [172]. More importantly, the clinical efficacy of Avastin (150 kDa) and Eylea (110 kDa) demonstrates that some penetration of larger molecules is both possible and sufficient to exert a therapeutic effect. At a minimum, the relationship between molecular size and efficacy is incompletely understood, especially when a part of the eye is compromised by the underlying pathology. There may be an optimum molecular size that represents a trade-off between efficient tissue penetration required for best efficacy, long residence time, and rapid systemic clearance once the drug leaves the eye. With molecular mass of about 50 kDa, Macugen, Fovista, Zimura, and Lucentis appear to be close to that optimum.

Since fully truncated aptamers are typically in the 8–12 kDa range, adjusting the overall molecular mass either by conjugation to inert moieties, such as PEG, or by multimerization is readily achievable. Assuming that metabolism is not the main mechanism for clearance, pharmacokinetic properties can be adjusted on the basis of molecular weight and residence times in animal models correlated with those observed in patients. While there are certainly nucleases in the eye, their amount in the vitreous humor is lower overall compared with that observed in plasma [173] (unsurprising given the fact that total protein concentration in vitreous humor is about 1% of that in plasma), so a less extensive protection against nucleases may be needed for most aptamer drug candidates.

Despite an excellent safety profile, frequent IVT injections carry a risk of sight-threatening adverse events such as endophthalmitis, retinal detachment, hemorrhage, and sustained increases in intraocular pressure [174]. Frequent office visits and occasional eye pain, sometimes severe, as a result of the injection procedure are a burden on the patient. Attempts to reduce the number of injections of anti-VEGF agents have led to inferior outcomes [175]. Now with the potential for polypharmacy through IVT injection, such risks and burdens will only increase.

One possibility to reduce the burden of IVT injections is the development of sustained release formulations. Such formulations could take the form of nanoparticles or microparticles injected into the vitreous or polymer-based dosage forms implanted into or near the vitreous cavity. This is challenging with protein-based drugs that can denature and aggregate during the formulation process [176]. Denatured or aggregated proteins are less potent and may be more immunogenic [177], which can lead to serious clinical consequences [178].

Aptamers, on the other hand, have low immunogenic potential and can tolerate a comparatively wide temperature and pH range. Even upon exposure to organic solvents, aptamers readily refold when the solvent is removed. In fact, aptamers are routinely purified at high temperatures (up to 80°C) in the presence of organic solvents such as ethanol and acetonitrile. Such robust physical properties make aptamers an ideal choice for creating sustained release formulations and may be a future direction for aptamer therapeutics in the eye.

What are the other opportunities for aptamer-based therapeutics in the eye? Over the past decade, we have focused our attention on improving the performance of aptamers by front loading nucleic acid libraries with functional groups absent in natural nucleic acids, but common in antigen-binding sites of antibodies and other protein–protein interaction surfaces. The 5-position of pyrimidines has been a suitable place for introducing such diversity-enhancing moieties through a conformationally restricted amide linkage [179]. These modifications have dramatically increased the success rate of SELEX with protein targets, broadening the scope of proteins that are accessible to SELEX [180]. To account for their composition and selection for stable complexes, we named this new generation of nucleic acid ligands slow off-rate modified aptamers or SOMAmers.

Based on cocrystal structures of SOMAmer-protein complexes, these modifications create entirely new structural motifs and generate extensive hydrophobic binding surfaces, in contrast to mainly polar surfaces observed with conventional aptamers. Among the SOMAmers we have discovered are novel nucleic acid ligands to some of the now clinically validated targets we reviewed here: VEGF, PDGF-BB, C5, and several other members of the complement pathway, such as C3, factor B, and factor D ([181] and unpublished results). As we have seen with PDGF-BB [168], SOMAmers usually have higher affinities and slower dissociation rates compared with conventional aptamers [180]. With VEGF, we have been able to identify high-affinity (subnanomolar) ligands to VEGF-121, which has previously been a very challenging target (unpublished results).

Some of these reagents may have the potential to be developed into therapeutic candidates. However, a substantial challenge in any drug development program has always been the identification of a molecular target or a pathway that represents a good drug target. AMD remains a highly complex disease and current treatments work well with many, but clearly not all, patients. Anti-VEGF therapy results in a visual acuity gain of three lines in about a third of the AMD patients. The addition of PDGF antagonists or C5 antagonists to anti-VEGF therapy increases the number of patients who respond well, but there is still considerable room for improvement.

We have recently developed a SOMAmer-based assay capable of simultaneously measuring more than 3,000 proteins with high precision and sensitivity. The assay works by transforming protein concentrations into a corresponding DNA signature, essentially taking advantage of the dual nature of SOMAmers as both folded binding entities with defined shapes and as unique nucleic acid sequences recognizable by specific hybridization probes [169]. In collaboration with Dr. Naresh Mandava at the University of Colorado, we are applying this assay as a high-content proteomic discovery tool to examine the changes in protein expression in the eyes of AMD patients in response to treatment with anti-VEGF drugs. The ability to identify patients who do or do not respond well to anti-VEGF therapy would be highly advantageous and this could lead to the development of a new companion diagnostic. As important is the possibility of identifying new targets for therapeutic intervention and eventually allowing a larger fraction of AMD patients to maintain their vision.

There remain many unmet medical needs in ophthalmology not only in AMD but also in many other conditions in both the posterior and the anterior chambers of the eye. For ocular disorders, aptamers represent a versatile class of molecules that can be used in a wide range of drug development phases, including target discovery, target validation, companion diagnostics, and ultimately therapeutics.

For aptamer therapeutics in general, success in ophthalmology carries a valuable lesson—not all indications make sense, but many do with compelling rationale. Treatment of conditions, where the drug can be delivered either locally (like in AMD) or by the parenteral route of administration and the ability for potent and specific antagonism is matched with unique tissue distribution and pharmacokinetic properties, represents such opportunities [182–186]. In terms of what is important to patients, few things are valued as highly as maintaining good vision.
